# Positive Expression of Human Cytomegalovirus Phosphoprotein 65 in Atherosclerosis

**DOI:** 10.1155/2016/4067685

**Published:** 2016-11-20

**Authors:** Zhe Wang, Jun Cai, Mingming Zhang, Xiaojing Wang, Hongjie Chi, Haijun Feng, Xinchun Yang

**Affiliations:** ^1^Department of Cardiology, Beijing Chaoyang Hospital, Capital Medical University, Beijing 100020, China; ^2^The First Cooperation of Chinese and Western Medicine Hospital of Beijing, Beijing, China

## Abstract

Previous studies showed that human cytomegalovirus (HCMV) is associated with atherosclerosis. However, local vascular atherosclerosis related HCMV infection and protein expression remain unclear. This study aimed to assess the relationship between HCMV infection and atherosclerosis. Formalin-fixed, paraffin-embedded peripheral artery specimens were obtained from 15 patients with atherosclerosis undergoing vascular surgery from 2008 to 2010 at Zhongnan Hospital, Wuhan University. Pathological analyses were carried out after hematoxylin and eosin (H&E) and Masson trichrome staining.* In situ *hybridization and immunohistochemistry with two different monoclonal antibodies were employed to detect HCMV nucleic acids and proteins, respectively. H&E and Masson trichrome staining showed homogeneous extracellular matrix in femoral artery, while smooth muscle fibers were interlaced with collagen fibers; in carotid artery, inflammatory cell infiltration, foam cell vascular change, cholesterol crystals, and layered collagen fibers were observed.* In situ* hybridization showed no expression of HCMV nucleic acids in all 15 cases. Immunohistochemical staining for protein immediate-early protein (IE1 72) was negative in all cases, while phosphoprotein 65 (pp65) expression was detected in 14 cases. A high rate of positive pp65 signals was found in patients with atherosclerosis, suggesting that local HCMV infection may be associated with the pathogenesis of atherosclerosis. Further studies on this relationship are warranted.

## 1. Introduction

Atherosclerosis, an inflammatory artery disease characterized by alterations in the levels of lipids and other metabolites, is the most important cause of cardiovascular diseases, including stroke, myocardial infarction, heart failure, and arterial aneurysm; it is also the primary cause of chronic renal failure [1, 2]. Atherosclerotic cardiovascular disease (ACD) comprises ischemic heart disease (IHD) and ischemic stroke [3]. ACD caused 247.9 deaths/100,000 individuals in 2013 worldwide, which represents 84.5% of cardiovascular deaths and 28.2% of all-cause mortality [4]. It is caused by the combined effects of multigenetic, environmental, and other factors [5]. However, the pathogenesis and etiologic factors of atherosclerosis are not completely understood.

Several studies have linked atherosclerosis to infection by viruses such as hepatitis C [6, 7], human immunodeficiency virus (HIV) [8, 9], and cytomegalovirus (CMV) [10, 11]. Human cytomegalovirus (HCMV) causes the most common perinatal viral infection in industrialized countries, affecting about 1% of all newborns in the United States [12]. HCMV, also known as human herpes virus-5 (HHV-5), has a 240 kb genomic DNA and is strictly species-specific for humans [13]. Histologically, HCMV infected tissues show cell swelling, multinucleated giant cells, and nuclear inclusions.* In vitro *transfection assays pointed to a possible p53 inactivation by IE1 72, an HCMV immediate-early gene product, in restenosis [14]. However, few direct evidences of an association of HCMV infection with endothelial dysfunction and atherosclerosis have been published, with some reports reaching disparate conclusions [15–17]. Previous studies also showed that plasma HCMV IgG or anti-CMV antibody level is associated with atherosclerosis [18–20]. However, direct evidence of HCMV involvement in atherosclerosis remains unclear. In this regard, an interesting question is whether local vascular atherosclerosis correlates with HCMV protein expression.

Therefore, this study aimed to assess the relationship between HCMV infection and atherosclerosis. Interestingly, we found a high rate of positive pp65 signals in patients with atherosclerosis, indicating a possible role for HCMV in the pathogenesis of atherosclerosis. Our findings provide a basis for further understanding of atherosclerosis pathogenesis, prevention, and treatment.

## 2. Materials and Methods

Peripheral arterial specimens were collected from 15 patients (13 men and 2 women; mean age of 68.4 years, from 51 to 110 years) with atherosclerosis undergoing vascular surgery from 2008 to 2010 at Zhongnan Hospital, Wuhan University. Patients older than 18 years, diagnosed with atherosclerosis (including coronary heart disease, artery stenosis, and artery obliterans), and undergoing vascular surgery (including endarterectomy, artery shunt, and arterial bypass) were included in this study. Patients with severe heart, lung, liver, and kidney dysfunction, distal artery stenosis, or arterial occlusion diagnosed by Digital Subtraction Angiography (DSA) were excluded.

### 2.1. Diagnosis [21]

Coronary heart disease (CHD) was diagnosed by coronary angiography. The Judkins method was used to perform right and left coronary angiography to evaluate the degree of stenosis of the main, circumflex, and anterior descending branches of the left coronary artery, as well as the main and other branches of the right coronary artery. Diameters were measured by visualization to determine the degree of stenosis, and CHD was diagnosed with ≥50% stenosis.

Hypertension was diagnosed as follows: in the absence of pressure lowering drugs, blood pressure was measured thrice on different days. Systolic blood pressure ≥ 140 mmHg and/or diastolic blood pressure ≥ 90 mmHg (WHO/ISH diagnostic criteria, 1999) indicated hypertension.

Diabetes was diagnosed according to clinical manifestations and blood glucose (plasma glucose ≥ 11.1 mmol/L [200 mg/dL] at any time point, or fasting plasma glucose ≥ 7.0 mmol/L [126 mg/dL], or plasma glucose ≥ 11.1 mmol/L [200 mg/dL] at 2 h after oral glucose tolerance tests [OGTT]) according to WHO diagnostic criteria published in 1999.

Dyslipidemia was diagnosed with total fasting serum cholesterol ≥ 5.18 mmol/L, triglyceride ≥ 1.70 mmol/L, or low-density lipoprotein cholesterol ≥ 3.37 mmol/L, according to the Chinese Guideline for the Prevention and Treatment of Dyslipidemia (2007).

Arterial stenosis and artery obliterans were evaluated by DSA, including serious artery calcification, elongation, vascular lumen distortion, diffuse irregular “worm like” stenosis, or segmental occlusion.

### 2.2. Tissue Collection

Peripheral artery tissues with a mean length of 27.4 ± 1.1 mm were collected during surgery, fixed in neutral buffered formalin, and paraffin embedded. To assess basic tissue composition and structure, all specimens were submitted to hematoxylin and eosin (H&E) and Masson trichrome staining.* In situ *hybridization (ISH) and immunohistochemistry (IHC) were used to detect HCMV and its distribution in tissue samples.

### 2.3. Paraffin Embedding and H&E Staining

Specimens were fixed in neutral buffered formalin for 24 h and embedded in paraffin at 65°C. Sections were cut at a thickness of 4–6 *μ*m, with three to four sections placed on each slide (FRC-05, Matsunami Glass Industry Co., Ltd., Japan). Sections were then baked for 16 h at 56–60°C and stored at room temperature.

After 2 h baking at 50°C, sections were deparaffinized twice in xylene (15 min each), immersed in 95% alcohol for 1 min followed by 70% alcohol for 1 min, washed in running tap water three times, and stained with Harris hematoxylin solution for 10 min. Sections were then placed under running tap water for 2 min, treated with 1% acid alcohol for 10 sec, washed with running tap water for 2 min, counterstained with 1% eosin solution for 2 min, rinsed under running tap water for 1 min, dehydrated in graded ethanol, cleared twice in xylene (15 min each), and mounted with neutral gum.

### 2.4. Masson Trichrome Staining

Samples were baked for 2 h at 50°C, deparaffinized twice in xylene (15 min each), immersed in graded ethanol (100%, 100%, 95%, 95%, 90%, 80%, 70%, 60%, and 50%, each for 1 min), washed with running tap water three times, fixed with saturated picric acid and 3% mercuric chloride for 40 min, and rinsed with running tap water for 10 min. Then, samples were immersed in 0.5% acetic acid for 1 sec, acid fuchsine : Ponceau 2R (1 : 2) for 5 min, 0.5% acetic acid for 1 sec, and 1% phosphomolybdic acid for 2 sec. This was followed by counterstaining with brilliant green for 3 min, 0.5% acetic acid for 2 sec, and 100% ethanol twice (each for 2 sec). Finally, samples were rinsed twice in xylene (15 min each) and mounted with neutral gum.

### 2.5. Immunohistochemistry

Tissue sections were deparaffinized as described above for Masson trichrome staining. After endogenous peroxidase blocking, the samples were incubated with primary antibodies, including anti-cytomegalovirus pp65 (1 : 100; ab49214, Abcam, Cambridge, UK), anti-cytomegalovirus IE1 72 (1 : 100; ab65104, Abcam), and anti-beta-actin (1 : 100; TA-09, ZSGB- BIO, Beijing, China) antibodies, at 4°C overnight. Next, rabbit anti-mouse HRP secondary antibodies (IgG H&L) (1 : 300; ab6728, Abcam) were added for 2 h at room temperature. The DAB chromogenic solution was added for detection for 3–20 minutes at room temperature, monitoring staining intensity under a light microscope. Finally, counterstaining was performed with hematoxylin.

### 2.6. *In Situ *Hybridization

Sections were dewaxed by baking for 2 h at 50°C and deparaffinized twice in xylene (15 min each), immersed in 100% ethanol for 5 min, and air dried for 10 min. An appropriate stomach enzyme solution was placed on tissue slices and incubated for 30 min at 37°C; then, specimens were dehydrated with graded ethanol (70%, 95%, and 100%). Three probes were chosen for ISH: the RISH positive control DIG probe (Q152P.9900, PanPath Company, AC, Netherland) was used as a positive control (composed of a mixture of oligonucleotides complementary to the poly-A tail of mRNA, supplied as stable ready-to-use reagents labelled with digoxigenin); detection probe was obtained from a HCMV nucleic acid* in situ* hybridization kit (ISH-5036, PanPath Company, Netherland); RISH negative control DIG probe (Q101P.9900, PanPath Company), a negative control probe for RNA, was used as negative control. Sections were then rinsed with PBS for 10 minutes. Appropriate anti-digoxin antibodies (DIG-HRP REMBRANDT® CMV DISH kit, A200 K.9901, PanPath Company) were added to tissue slices, incubated at 37°C for 30 minutes, and washed in PBS three times (2 min each). Tissue sections were then counterstained using Mayor hematoxylin for 5–10 seconds, rinsed with distilled water, and dehydrated in graded ethanol. Specimens were finally vitrified in xylene twice (15 min each) and sealed with neutral gum.

## 3. Results and Discussion

### 3.1. Characteristics of Patients with Atherosclerosis

A total of 15 patients were assessed, including 13 males and 2 females; they were 68.4 ± 14.3 years old. Hypertension, type 2 diabetes mellitus, hyperlipidemia, coronary heart disease, carotid artery stenosis, subclavian artery stenosis, vertebral artery stenosis, peripheral arterial disease, aortic and iliac artery obliterans, and renal artery obliterans were diagnosed in 5 (33.3%), 4 (26.7%), 2 (13.3%), 3 (20.0%), 8 (53.3%), 1 (6.7%), 1 (6.7%), 3 (20.0%), 1 (6.7%), and 1 (6.7%), respectively. Almost all patients were diagnosed with more than one pathology. Surgical methods included carotid endarterectomy (9, 60.0%), carotid-subclavian artery shunt (1, 6.7%), and arterial bypass (5, 33.3%). Samples were collected from carotid (10, 66.7%), subclavian (1, 6.7%), femoral (3, 20.0%), and iliac (3, 20.0%) arteries. Femoral and iliac artery samples were both collected from 2 patients with peripheral arterial disease. Detailed patient characteristics and sample collection sites are provided in Table 1.

### 3.2. Histological Features of Atherosclerosis

**Figure 1:**
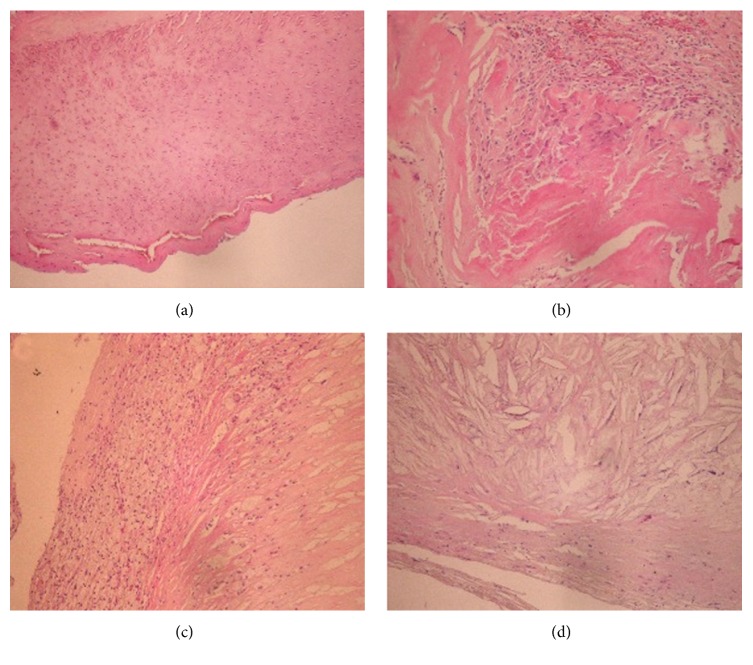
H&E stained peripheral artery specimens. Samples were submitted to H&E staining, and representative sections are shown. (a) Homogeneous femoral artery and extracellular matrix (magnification ×100). (b) Carotid artery infiltrated by vascular smooth muscle cells and inflammatory cells (×400). (c) Carotid artery showing foam cells (×100). (d) Carotid artery containing cholesterol crystals dispersed in the tissue (×400).

**Figure 2:**
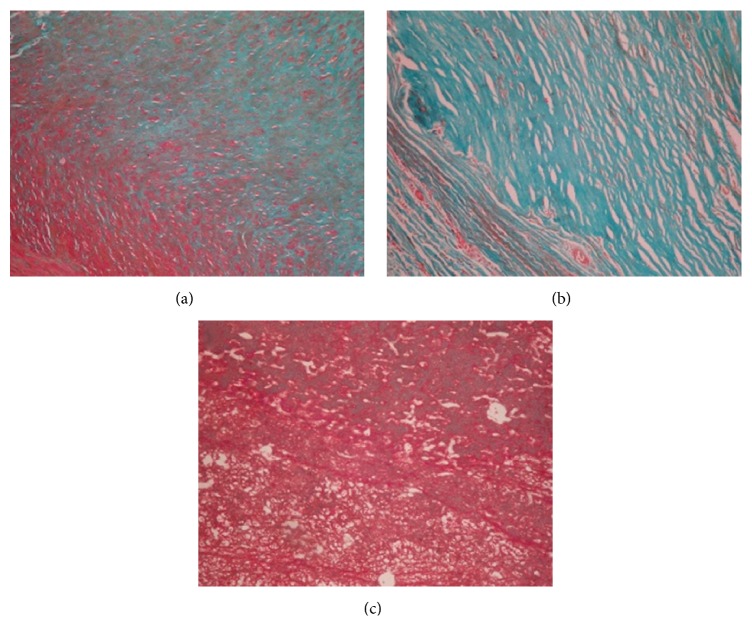
Masson trichrome stained peripheral artery specimens. (a) Femoral artery with smooth muscle tissue and collagen fibers interlaced (×400). (b) Carotid artery with layered collagen fibers (×400). (c) Carotid artery with smooth muscle fibers, some showing foamy change (×400). Green, collagen fibers; red, muscle fibers; pale red, epithelial cells; orange, red blood cells.

Representative H&E staining data are presented in Figure 1, and the observed pattern was the same in all cases. Homogeneous femoral artery and extracellular matrix were observed (Figure 1(a)); meanwhile, carotid artery was infiltrated by vascular smooth muscle fibers and inflammatory cells (Figure 1(b)). Foam cells (Figure 1(c)) and cholesterol crystals (Figure 1(d)) were observed as well. Masson trichrome staining revealed smooth muscle fibers interlaced with collagen fibers (Figure 2(a)). In carotid artery, foam cell vascular change and layered collagen fibers were observed (Figures 2(b) and 2(c)).

### 3.3. Absence of HCMV Acid Nucleic Detection in the Tissues

**Figure 3:**
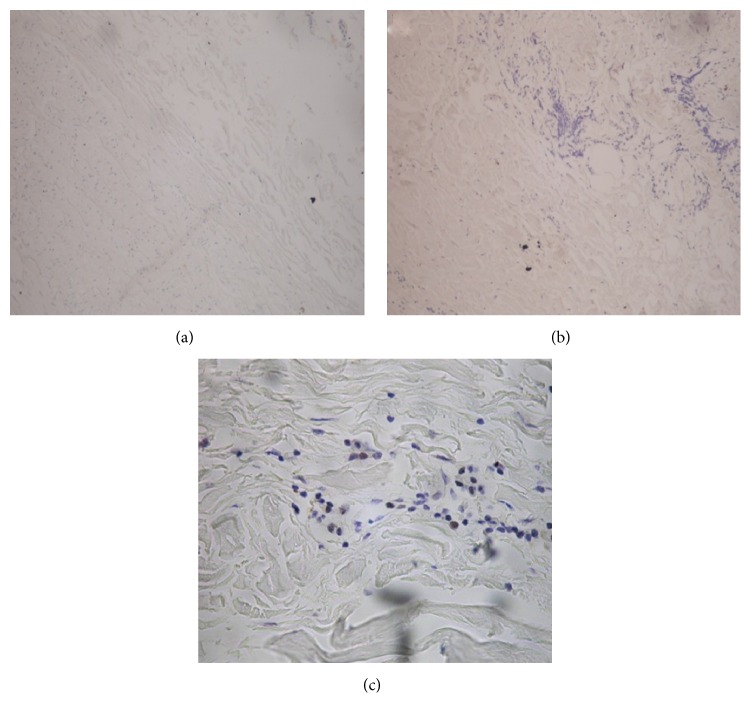
HCMV nucleic acid detection by* in situ *hybridization in peripheral arterial specimens. (a) HCMV nucleic acid was absent in all patient specimens (×400). (b) Negative control (×400). (c) Positive control (×400).

All specimens were negative for HCMV nucleic acid expression. Indeed, no positive signals were found in the cell nucleus or cytoplasm of the full-thickness vascular wall (Figure 3(a)). Expression was not obtained for the PBS buffer negative control sample (Figure 3(b)); meanwhile, positive control showed positive brown signals in the nucleus (Figure 3(c)).

### 3.4. HCMV Viral Protein Detection in Arteriosclerotic Tissues

**Figure 4:**
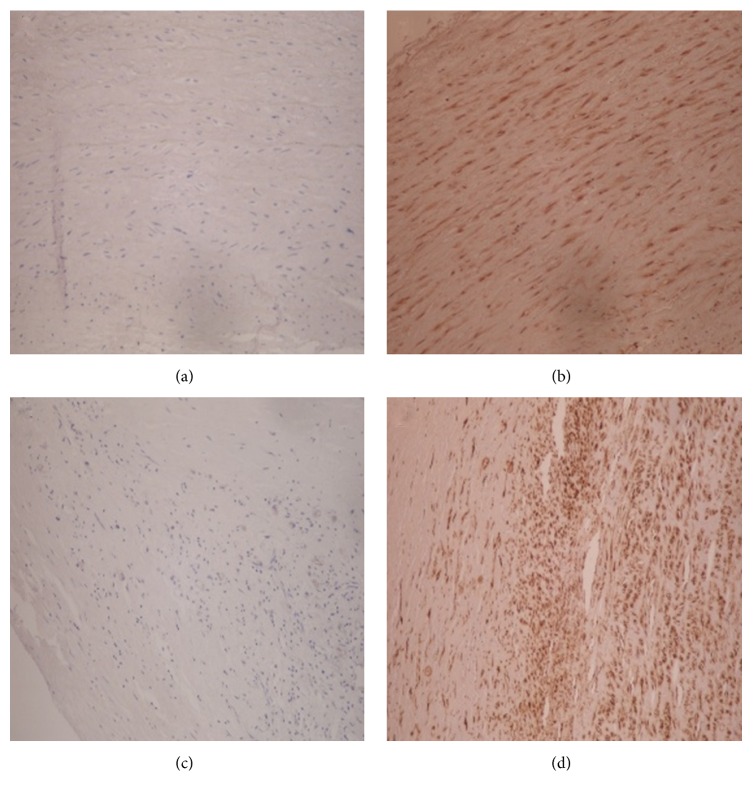
Immunohistochemical staining of peripheral arterial specimens. (a) No expression of the HCMV protein IE1 72 (×400). (b) Positive expression of the HCMV protein pp65 (×400). (c) No signal with PBS (control) (×400). (d) Positive expression with *β*-actin antibody (positive control) (×400).

The HCMV protein IE1 72 was absent in all 15 cases (Figure 4(a)). Meanwhile, human cytomegalovirus protein pp65 was detected in 14 cases, as scattered brown immunoreactive signals in vascular endothelial cells and smooth muscle fibers (Figure 4(b)). One case was negative for pp65. Negative staining was obtained with PBS (negative control) as shown in Figure 4(c); meanwhile, HCMV *β*-actin stained positive in the cytoplasm with the positive control antibody (Figure 4(d)).

Using ISH and immunohistochemistry, we demonstrated that peripheral artery specimens do not express HCMV nucleic acids or the IE1 72 viral protein; however, another viral protein, pp65, was detected in almost all samples (14/15).

As shown above, HCMV nucleic acid expression was negative in all 15 peripheral artery specimens examined. Meanwhile, positive and negative controls indicated the reliability of this method. Our data could be explained by the following. First, HCMV levels in the peripheral artery tissue may be below the detection limit of the ISH method; alternatively, atherosclerosis causes endothelial tissue damage, reducing the number of endothelial cells, for which HCMV has a tropism; this would result in too low viral DNA content in the tissue and consequently no viral DNA detection. Therefore, ISH may be not sensitive enough for HCMV detection in these tissues. Indeed, a recent study showed that, for enterovirus detection, RT-PCR was the most sensitive technique, followed by LC/MRM/MS/MS and IHC, while ISH turned to be the least sensitive method [22]. Secondly, the patients analyzed in this study indeed had HCMV infection, but involvement of the peripheral artery tissue is inconsistent and the assessed samples were inadequate to detect HCMV presence by ISH. That is, we may have missed HCMV in some patients due to small sample size.

According to order of expression, the HCMV genome is divided into immediate early (IE), early (E), and late (L) genes, which are sequentially expressed. We found that IE1 72 viral was absent in all samples assessed in this study. The IE1 protein is the first DNA-binding nuclear protein produced by the virus; its main function is to activate viral gene expression. IE1 also interferes with intracellular pathways, including gene regulation, cell cycle progression, signal transduction, apoptosis, and interaction of specific nuclear areas [23]. IE1 is a negative regulator of host immune response. It inhibits cellular immune responses via prevention of interferon-sensitive gene expression.

Interestingly, the pp65 protein was detected in almost all peripheral artery specimens. This was not surprising since pp65 is the major component of mature HCMV particles [24]. In addition, the pp65 gene is expressed during both the early and late periods of the infection cycle [25]; its product is a HCMV tegument protein that induces specific cellular immune response* in vitro *and* in vivo *[26, 27]. Our data suggest a possible involvement of pp65 in atherosclerosis.

Of note, cytoplasmic expression of *β*-actin was detected and no signal was found with the PBS buffer control, indicating the reliability of the IHC staining procedure employed here. A few limitations of the present study should be mentioned. First of all, we assessed only 15 patients, indicating a relatively small sample size. In addition, we did not include HCMV without cardiovascular diseases as controls. Furthermore, it would be useful to test patients with different atherosclerosis disease stages, in order to fully understand the role of HCMV in the development and progression of this cardiovascular disease. Finally, more sensitive methods should be used for virus detection, including RT-PCR, LC/MRM/MS/MS, and IHC. Therefore, further in-depth studies addressing these issues are warranted to confirm our findings.

## 4. Conclusions

Overall, IHC analyses of peripheral artery samples from 15 patients demonstrate an association of HCMV infection with atherosclerosis. Our findings provide a basis for further understanding of the pathogenesis, prevention, and treatment of atherosclerosis.

## Figures and Tables

**Table 1:** Patient basic information and sample drawn position.

Patient number	Gender	Age (years)	Clinical diagnosis	Sample drawn position
1	Female	73	Hypertension, coronary heart disease, bilateral carotid artery stenosis	Carotid artery
2	Male	63	Hypertension, coronary heart disease, bilateral carotid atherosclerosis	Carotid artery
3	Male	63	Hypertension, right carotid artery stenosis, type 2 diabetes	Carotid artery
4	Female	73	Hypertension, left carotid artery stenosis	Carotid artery
5	Male	79	Hypertension, right subclavian artery stenosis, cervical spondylosis	Subclavian artery
6	Male	60	Right carotid artery stenosis, type 2 diabetes	Carotid artery
7	Male	80	Peripheral arterial disease	Iliac artery, femoral artery
8	Male	70	Peripheral arterial disease	Iliac artery, femoral artery
9	Male	110	Left carotid artery stenosis	Carotid artery
10	Male	61	Left carotid stenosis, right vertebral artery stenosis	Carotid artery
11	Male	65	Left carotid stenosis, hyperlipidemia, bronchial asthma	Carotid artery
12	Male	56	Aortic-iliac artery obliterans, bilateral renal artery obliterans, coronary heart disease, type 2 diabetes, hyperlipidemia	Iliac artery
13	Male	55	Peripheral arterial disease	Femoral artery
14	Male	51	Right carotid artery aneurysm, duodenal ulcer	Carotid artery
15	Male	67	Right carotid artery stenosis, type 2 diabetes	Cervical spinal Decompression Surgery
